# The value of quantitative fluorescence polymerase chain reaction for the products of conception in the era of copy number variation sequencing

**DOI:** 10.3389/fgene.2025.1750362

**Published:** 2026-01-09

**Authors:** Shaozhe Yang, Hewei Zhang, Yongli Wang, Qingwei Zhang, Yongyong Wu, Xiuhong Fu, Rongxiang Li

**Affiliations:** 1 Henan Key Laboratory of Fertility Protection and Aristogenesis, Luohe Central Hospital, Luohe, China; 2 Luohe Reproductive Medicine and Genetics Center, Luohe Central Hospital, Luohe, China; 3 Animal Diseases and Public Health Engineering Research Center of Henan Province, Luoyang, China; 4 Gynecology and Obstetrics Department, Luohe Central Hospital, Luohe, China

**Keywords:** chromosome aberrations, copy number variation sequencing, products of conception, quantitative fluorescent polymerase chain reaction, spontaneous miscarriage

## Abstract

**Purpose:**

The purpose of this research was to assess the effectiveness of copy number variation sequencing (CNV-Seq) and quantitative fluorescence polymerase chain reaction (QF-PCR) in detecting chromosomal abnormalities in products of conception of women and to evaluate the pros and cons of three different combined detection strategies, CNV-Seq and QF-PCR.

**Methods:**

Genetic testing was conducted on 701 samples using QF-PCR and CNV-Seq. The detection efficiency of the two methods for various chromosomal abnormalities was calculated. The relationships between maternal age, miscarriage gestational age, number of prior miscarriages, and conception method with fetal chromosomal abnormalities were assessed. The detection efficiency and cost of three different combined detection strategies were compared.

**Results:**

Maternal cell contamination was found in 2.57% of samples. The overall occurrence rate of various chromosomal abnormalities was as high as 67.20% (459/683). QF-PCR was effective in detecting maternal cell contamination, triploidy, and common aneuploidies but was not effective in detecting mosaicism. Advanced maternal age, abnormal pregnancy history, early abortion, and naturally conceived products of conception were more likely to detect aneuploidy but were unrelated to triploidy and CNVs.

**Conclusion:**

QF-PCR can effectively complement the limitations of CNV-Seq. Employing QF-PCR to rule out maternal cell contamination prior to performing CNV-Seq on all samples is the best detection strategy.

## 1 Introduction

Spontaneous abortion refers to the natural loss of clinically recognized pregnancies before the fetus reaches viability. Approximately 15%–20% of pregnancies that are clinically recognized result in pregnancy loss (Tarleton et al., 2024), with the majority occurring in the early stages of pregnancy (Alwazzan et al., 2020). Spontaneous abortion before 12 weeks of pregnancy is known as early abortion, whereas spontaneous abortion between 12 and 28 weeks is called late abortion (Dou et al., 2023). Factors leading to spontaneous abortion include genetic factors, anatomical abnormalities, endocrine disorders, immune diseases, etc., (Gui et al., 2022). Fetal chromosomal abnormalities are responsible for more than 50% of spontaneous abortions, making them the most common cause (Andreescu et al., 2023).

Chromosomal aneuploidy, triploidy, and pathogenic copy number variations are the main genetic factors causing spontaneous abortion (Smits et al., 2020; Yang et al., 2024). Chromosome karyotyping, a traditional technique for embryo chromosomal examination, is now rarely used due to its high failure rate, time-consuming process, low resolution, and need for fresh and uncontaminated samples (Sahoo et al., 2017; Zhang et al., 2018). Copy number variation sequencing (CNV-Seq), which uses next-generation sequencing, offers high throughput, high resolution, low cost, and low sample requirements (Shi et al., 2023). It can effectively detect mosaicism at rates as low as 10% (Shi et al., 2023), making it a frontline method for genetic testing of products of conception in recent years. CNV-Seq has enhanced the detection of submicroscopic deletions and duplications, thereby improving the identification of genetic etiologies for miscarriage (Xue et al., 2023; Shao et al., 2024). However, the increased resolution of these methods also leads to the frequent identification of copy number variations (CNV) that are benign polymorphisms or of uncertain clinical significance, presenting a challenge for clinical interpretation and genetic counseling (Zhao and Fu, 2019).

**FIGURE 1:**
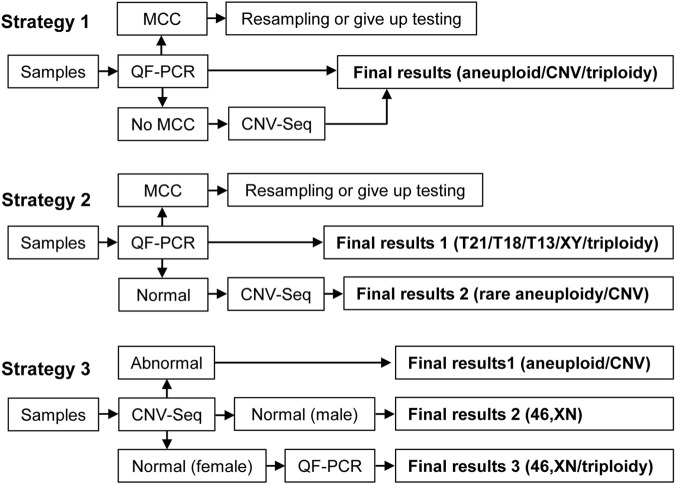
Three different testing strategies. MCC, maternal cell contamination.

The main drawback of CNV-Seq for genetic testing of products of conception is its inability to detect triploidy (Dai et al., 2024). Quantitative fluorescence polymerase chain reaction (QF-PCR) based on short tandem repeats can not only detect triploidy at a low cost but also determine whether products of conception contain maternal cell contamination. Previous studies have shown that combining QF-PCR with CNV-Seq can increase the detection rate of chromosomal abnormalities and effectively identify samples with maternal cell contamination (Chen et al., 2023; Chen et al., 2024; Dai et al., 2024). The detection strategy for the combined use of QF-PCR and CNV-Seq in the literature is inconsistent. In Chen’s study (Chen et al., 2021), QF-PCR was first used to exclude maternal cell contamination in all samples, followed by CNV-Seq detection of samples without maternal cell contamination (Strategy 1). In Donaghue’s research (Donaghue et al., 2017), only samples with no abnormalities on QF-PCR were subjected to CNV-Seq testing (Strategy 2). In Chen’s study (Chen et al., 2023) and Kato’s study (Kato et al., 2022), all samples were first tested using CNV-Seq, followed by the use of QF-PCR to determine whether the samples identified as normal females by CNV-Seq were triploid (Strategy 3). The detection process of the three strategies is shown in Figure 1. Combining QF-PCR with CNV-Seq is meaningful, but different detection strategies correspond to different detection efficiencies and costs.

In this study, the detection results of 683 products of conception tested with CNV-Seq and QF-PCR were compared, detailing the detection efficiency of the two techniques for various chromosomal abnormalities and the detection efficiency and cost changes of the two techniques when combined under different testing strategies.

## 2 Methods and materials

### 2.1 Subjects

This retrospective study included 701 women who underwent products of conception genetic testing at Luohe Central Hospital in China from January 2022 to October 2024. The inclusion criteria were as follows: (1) spontaneous abortion; (2) singleton pregnancy. This study adhered to strict privacy protection principles and was approved by the Medical Ethics Committee of Luohe Central Hospital (No. MEC-2021-016, approval date 3 November 2021).

### 2.2 Sample isolation and DNA extraction

The products of conception were rinsed with sterile saline solution three times to completely remove any blood from their surface. Trained personnel extracted approximately 20 mg of tissue from the products of conception and chopped it up. The tissue DNA was extracted via the QIAamp DNA Mini Kit (Qiagen, NY, United States). If the QF-PCR results indicate maternal cell contamination, a second sample isolation should be performed. During the sampling process, if the sample contains chorionic villi, utilize chorionic villus testing. If the chorionic villus was fetal tissue, skin should be taken for testing. If the tissue was indistinguishable, sample at any location and compare the results with the mother’s QF-PCR test simultaneously.

### 2.3 QF-PCR

QF-PCR was performed according to the manufacturer’s instructions using the 21, 18, 13, and sex chromosome aneuploidy detection kits produced by Shanghai Cubicise Medical Co., Ltd., China. This kit can detect a total of 30 genetic markers located on chromosomes 21, 18, 13, X, and Y. After multiple rounds of PCR, the PCR products were separated on an ABI 3500 capillary genetic analyzer (Applied Biosystems, Foster City, CA, United States) and analyzed using GeneMapper 6.0 software (Applied Biosystems, Foster City, CA, United States).

### 2.4 CNV-seq

Using a CNV detection kit and a next-generation sequencing library construction kit (Berry Genomics, Beijing, China), CNV library construction, purification, and quality control were performed. Next-generation sequencing and bioinformatics analysis were performed using a NextSeq CN500 sequencer (Illumina, San Diego, California, United States). The human reference genome sequence version GRCh37 was selected, and sequencing data were analyzed using the CNV detection algorithm developed by Tattini et al. (2015), which detects CNVs with a resolution of 100 kb. The pathogenicity of CNVs was assessed by referencing public databases. The clinical significance of CNVs is classified into five levels: benign, likely benign, variants of uncertain significance, likely pathogenic and pathogenic. In this study, pathogenic, likely pathogenic, and variants of uncertain significance were considered chromosomal abnormalities.

### 2.5 Statistical analysis

Data analysis was conducted using IBM SPSS 25.0 software (IBM Corporation, Armonk, New York, United States). Continuous variables are represented as the mean ± standard deviation (X ± S); categorical variables are represented as “n (%)”. Group comparisons were carried out using either the chi-square test or Fisher’s exact probability test, with statistical significance defined as *p* < 0.05. When comparing the efficiency of QF-PCR and CNV-Seq for detection, any abnormal cases identified by either QF-PCR or CNV-Seq were considered true positive cases. Products of conception with multiple types of chromosomal abnormalities, such as triploidy and aneuploidy, were included in different groups for repeated statistical analysis.

## 3 Results

### 3.1 Specimen characteristics in this study

**FIGURE 2:**
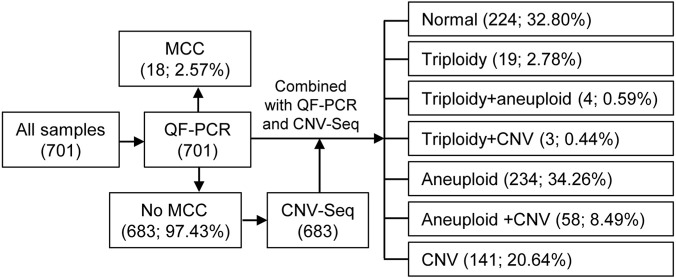
Flowchart of the products of conception samples tested in this study. MCC, maternal cell contamination.

The workflow of this study is shown in Figure 2. Among all 701 products of conception, 18 samples were found to have maternal cell contamination through QF-PCR and did not undergo further testing. The remaining 683 samples underwent CNV-Seq, including 523 chorionic villus samples, 74 fetal tissue samples, and 86 samples with unknown tissue. The basic information of the women and their products of conception samples can be found in Table 1. The mean maternal age was 31.22 ± 4.74 years (range: 18–45 years), with 163 cases (23.87%) in the advanced maternal age group (35 years old and above) and 520 cases (76.13%) in the younger maternal age group (below 35 years old). The mean miscarriage gestational age was 9.75 ± 4.07 weeks (range: 5–28 weeks), with 608 cases of early abortion (89.02%) and 75 cases of late abortion (10.98%). A total of 325 (47.58%) of the women had not previously experienced spontaneous abortion, 216 (31.63%) had experienced one spontaneous abortion, and 142 (20.79%) had experienced at least two spontaneous abortions. A total of 599 (87.70%) women conceived naturally, whereas 84 (12.30%) conceived through *in vitro* fertilization.

**TABLE 1:** Baseline characteristics of pregnant women/samples.

Characteristics	Number	Proportion (%)
Total samples	701	NA
Maternal cell contamination samples	18	2.57
No maternal cell contamination samples	683	97.43
Chorionic villi	523	76.57
Fetal tissue	74	10.83
Unknown sample	86	12.59
Maternal age	31.22 ± 4.74	NA
Age<35	520	76.13
Age≥35	163	23.87
Miscarriage gestational weeks	9.75 ± 4.07	NA
Early abortion (gestational age≤ 12 weeks)	608	89.02
Late abortion (12 weeks < gestational age ≤28 weeks)	75	10.98
Number of prior miscarriages		
0	325	47.58
1	216	31.63
≥2	142	20.79
Conception method		
Natural conception	599	87.70
*In vitro* fertilization	84	12.30

NA, not applicable.

### 3.2 Distribution of abnormal results in QF-PCR/CNV-seq

After conducting QF-PCR combined with CNV-Seq testing on 683 samples, chromosomal abnormalities were detected in 459 cases (67.20%), with only 32.80% of samples showing no chromosomal abnormalities, as shown in Figure 2. Out of 683 samples, 19 samples (2.78%) showed triploidy alone, 4 samples (0.59%) exhibited triploidy along with aneuploidy, and 3 samples (0.44%) displayed triploidy combined with CNV. Additionally, 234 samples (34.26%) solely presented chromosomal aneuploidy, 58 samples (8.49%) showed chromosomal aneuploidy along with CNV, and 141 samples (20.64%) indicated CNV alone.

### 3.3 Comparison of the detection efficiency of CNV-Seq and QF-PCR

Table 2 displays the detection performance of QF-PCR and CNV-Seq for 459 true positive cases out of 683 samples. Both QF-PCR and CNV-Seq were effective in detecting trisomy 21/trisomy 18/trisomy 13. QF-PCR identified all trisomy cases, while CNV-Seq only detected 50% (13/26) of them. In the detection of sex chromosome aneuploidy, QF-PCR identified 58 out of 66 cases, while CNV-Seq detected all 66 cases. The sex chromosome aneuploidy additionally detected by CNV-Seq were all cases of mosaicism, indicating that CNV-Seq is more effective in detecting mosaicism compared to QF-PCR. Due to the limitations of the reagent kit used in this study for detecting chromosomal aneuploidies and CNVs other than those of chromosomes 21, 18, and 13, CNV-Seq identified an additional 183 cases of autosomal trisomy and 4 cases of monosomy other than trisomy 21/trisomy 18/trisomy 13.

**TABLE 2:** Comparison of the detection efficiency of chromosomal abnormalities according to QF-PCR/CNV-Seq.

Abnormal types	TP	QF-PCR results				CNV-seq results			
		Positive		FPR	FNR	Positive		FPR	FNR
		n	%			n	%		
All abnormal results	459	132	19.33	28.76	71.24	449	65.74	97.82	2.18
Triploidy	26	26	3.81	100.00	0.00	13	1.90	50.00	50.00
Trisomy 21/Trisomy 18/Trisomy 13	50	50	7.32	100.00	0.00	50	7.32	100.00	0.00
Rare autosomal trisomy	183	0	0.00	0.00	100.00	183	26.79	100.00	0.00
Sex chromosome aneuploidy	66	58	8.49	87.88	12.12	66	9.66	100.00	0.00
CNV	202	0	0.00	0.00	100.00	202	29.58	100.00	0.00
Autosomal monomer	4	0	0.00	0.00	100.00	4	0.59	100.00	0.00

TP, true positive, Positive cases detected by QF-PCR, or CNV-seq, were considered true positive cases; FPR, false positive rate; FNR, false negative rate.

### 3.4 Relationships between clinical features and chromosomal abnormalities

The relationships between the rates of triploidy, aneuploidy (including autosomal trisomy, autosomal monosomy, and sex chromosome aneuploidy), and overall chromosomal abnormalities with maternal age, number of prior miscarriages, miscarriage gestational age, and conception method were compared on the basis of the results of QF-PCR combined with CNV-Seq.

Table 3 compares the detection rate of chromosomal abnormalities between the younger maternal age group and the advanced maternal age group, revealing a significant difference only in the aneuploidy detection rate (40.19% vs. 51.53%; *P* = 0.011), with no significant differences in other types of chromosomal abnormalities. Table 4 compares the detection rate of chromosomal abnormalities of three groups of women with 0, 1, and ≥2 prior miscarriages. The rates of aneuploidy and overall chromosomal abnormalities increased with the number of prior miscarriages, and the differences were statistically significant (*P* = 0.000; *P* = 0.000), whereas the differences in other indicators were not statistically significant. Table 5 compares the detection rate of chromosomal abnormalities from the women in the early abortion group and late abortion group. The results revealed that the detection rate of aneuploidy in the early abortion group was significantly greater than that in the late abortion group (45.32% vs. 27.03%; *P* = 0.003), whereas the other indicators were not significantly different. Table 6 compares the detection rate of chromosomal abnormalities among different conception methods. The aneuploidy and overall chromosomal abnormality rates were significantly greater in the natural conception group than in the *in vitro* fertilization conception group (45.24% vs. 29.76%; *P* = 0.007 and 68.95% vs. 54.76%; *P* = 0.010).

**TABLE 3:** Comparison of the distribution of chromosomal abnormalities detected by QF-PCR/CNV-seq according to maternal age.

Types	Age <35 (n = 520)		Age ≥35 (n = 163)		*P*
	n	%	n	%	
Triploid	17	3.27	8	4.91	0.331
Aneuploidy	209	40.19	84	51.53	0.011
CNV	154	29.62	46	28.22	0.733
Total abnormal sample	345	66.35	117	71.78	0.196

**TABLE 4:** Comparison of the distribution of chromosomal abnormalities detected by QF-PCR/CNV-seq according to the number of prior miscarriages.

Types	Number of prior miscarriages						*P*
	0 (n = 325)		1 (n = 216)		≥2 (n = 142)		
	n	%	n	%	n	%	
Triploid	10	2.99	9	4.37	7	4.93	0.595
Aneuploidy	112	33.43	93	45.15	91	64.08	0.000
CNV	83	24.78	68	33.01	51	35.92	0.059
Total abnormal samples	190	56.72	154	74.76	115	80.99	0.000

**TABLE 5:** Comparison of the distribution of chromosomal abnormalities detected by QF-PCR/CNV-seq according to the miscarriage gestational age.

Types	Miscarriage gestational weeks				*P*
	Early abortion (n = 609)		Late abortion (n = 74)		
	n	%	n	%	
Triploid	25	4.11	1	1.35	0.345
Aneuploidy	276	45.32	20	27.03	0.003
CNV	176	28.90	26	35.14	0.267
Total abnormal sample	413	67.82	46	62.16	0.328

**TABLE 6:** Comparison of the distribution of chromosomal abnormalities detected by QF-PCR/CNV-seq according to the conception method.

Types	Conception method				*P*
	Natural conception (n = 599)		In vitro fertilization (n = 84)		
	n	%	n	%	
Triploid	24	4.01	2	2.38	0.759
Aneuploidy	271	45.24	25	29.76	0.007
CNV	176	29.38	26	30.95	0.768
Total abnormal sample	413	68.95	46	54.76	0.010

### 3.5 Detection efficiency and economic cost of three different testing strategies

Table 7 compares the detection efficiency and overall cost of three detection strategies that combine QF-PCR with CNV-Seq, as shown in Figure 1. Strategy 1 is the detection strategy used in this study, whereas Strategy 2 and Strategy 3 are other detection strategies used in the literature. Both Strategy 2 and Strategy 3 reduce the number of positive detections and the cost of testing to some extent.

**TABLE 7:** Clinical value and economic cost of three different testing strategies.

Strategies	Strategy 1			Strategy 2			Strategy 3		
Detection efficiency	MCC	Chromosomal abnormality		MCC	Chromosomal abnormality		MCC	Chromosomal abnormality	
	n	n	%	n	n	%	n	n	%
	18	459	67.20	18	459	67.20	0	459	67.20
Total anomalies	634			596			634		
Number of QF-PCR	701			701			137		
Number of CNV-Seq	683			552			701		
Total expense(RMB)	2,309,280			1,973,920			1,904,160		

We assume that all 18 MCC, samples do not have any chromosomal abnormalities.

The cost of QF-PCR, is 800 RMB/sample; the cost of CNV-Seq is 2,560 RMB/sample.

MCC, maternal cell contamination.

Assuming that the 18 samples with maternal cell contamination detected by QF-PCR have no chromosomal abnormalities, the detection rate for chromosomal abnormalities in all three strategies would be 67.20% (459/683), with a total of 634 abnormalities detected via each strategy. Unlike Strategy 1 and Strategy 2, Strategy 3 fails to exclude the samples with maternal cell contamination. The total costs for the three strategies are 2,309,280 RMB, 1,973,920 RMB, and 1,904,160 RMB, respectively, on the basis of the costs of 800 RMB per sample for QF-PCR and 2,560 RMB per sample for CNV-Seq.

## 4 Discussion

Fetal chromosomal abnormalities are the main factor causing spontaneous abortion. Genetic testing of products of conception can determine whether the cause of spontaneous abortion is “genetic” or “nongenetic” and provide guidance for future reproduction. It is still necessary to compare the detection efficiency of CNV-Seq and QF-PCR for different types of chromosomal abnormalities and explore the advantages of different testing strategies. When choosing a detection strategy, priority should be given to the comprehensiveness of detection, followed by considering the low cost. In this study, we compared the detection efficiency of QF-PCR and CNV-Seq, and analyzed the detection efficiency and cost of different testing strategies. We also discussed the impacts of maternal age, miscarriage gestational age, number of prior miscarriages, and conception method on the detection rate of various types of chromosomal abnormalities.

In previous reports, the proportion of products of conception with chromosomal abnormalities was 58.95% (Chen et al., 2021), 61.2% (Wou et al., 2016), 60.82% (Chen et al., 2024), 54.6% (Dai et al., 2024), and 45.98% (Chen et al., 2023), respectively. In this study, the proportion of products of conception with chromosomal abnormalities was 67.20% (459/683), which is higher than that reported in the literature. This is mainly due to the inclusion of variants of uncertain significance in CNVs in our study.

The QF-PCR kit we use contains probes for detecting chromosomes 21, 18, 13, and X/Y, which are theoretically capable of detecting all cases of trisomy 21, trisomy 18, trisomy 13, sex chromosome aneuploidy, and triploidy. The results of this study showed that, with the exception of triploidy, the abnormalities identified by QF-PCR were confirmed via CNV-Seq, demonstrating that despite being used as a complement to CNV-Seq, QF-PCR is accurate and rapid in detecting common chromosomal aneuploidies. However, this study also identified shortcoming of QF-PCR: Its ability to detect mosaicism is limited, with QF-PCR identifying four cases of mosaic sex chromosome aneuploidy out of 683 samples, whereas CNV-Seq identified a total of 15 cases of mosaic sex chromosome aneuploidy. Previous studies have also shown that QF-PCR cannot detect low-level mosaicism (Qiao et al., 2022). In this study, the detection rate of maternal cell contamination was 2.57% (18/701). Despite the fact that sample isolation in this study was conducted by experienced laboratory personnel, the occurrence of maternal cell contamination could not be avoided. It is necessary to perform QF-PCR on maternal blood simultaneously to determine the presence of maternal cell contamination. In this study, CNV-Seq confirmed all chromosomal abnormalities discovered via QF-PCR except for triploidy, increasing the detection rate from 19.18% (131/683) with QF-PCR alone to 67.20% (459/683) with combined testing.

Previous studies have shown that maternal age, miscarriage gestational age, number of prior miscarriages, and conception method may influence the detection rate of chromosomal abnormalities in products of conception, but previous studies have not reached a consistent conclusion. Research has shown that in products of conception, the advanced maternal age group has a higher overall chromosomal abnormality rate than does the younger maternal age group (Gu et al., 2021; Zhang et al., 2021b). Several reports indicate that the detection rate of chromosomal abnormalities in the early abortion group is greater than that in the late abortion group, but the difference is mainly for aneuploidy (Chen et al., 2021; Chen et al., 2023). Various chromosomal abnormalities can result in pregnancy loss at different points during pregnancy (Chen et al., 2021). The relationship between an abnormal pregnancy history and fetal chromosomal abnormalities is still controversial (Chen et al., 2021; Zhang et al., 2021a; Gou et al., 2022). Prior studies have consistently indicated that the incidence of chromosomal abnormalities in in vitro fertilization pregnancies is generally lower than that in embryos conceived naturally (Pylyp et al., 2018; Wang et al., 2020). This is because the *in vitro* fertilization process helps reduce the occurrence of chromosomal abnormalities, such as avoiding polyspermy through intracytoplasmic sperm injection fertilization and selecting high-quality embryos for transfer (Rubio et al., 2003; Capalbo et al., 2022; Madjunkov et al., 2025). Our research revealed that advanced maternal age, a history of spontaneous abortion, early abortion, and natural conception are more likely to result in chromosomal aneuploidy in fetuses. However, there was no significant correlation of the detection rate of triploidy and CNVs with any factors tested.

The use of QF-PCR followed by CNV-Seq (Strategy 1) is a basic strategy for combined detection, but some studies have proposed optimized strategies to reduce the testing process and costs. The results of this study indicate that the types of products of conception chromosomal abnormalities are diverse and involve almost every chromosome. Among the 459 samples with chromosomal abnormalities, 132 samples were found to have multiple abnormalities, such as triploidy combined with aneuploidy and multiple aneuploidies. The causes of spontaneous abortion are diverse, and errors or omissions in the genetic testing of products of conception can lead to incorrect treatment directions. Priority should be given to the accuracy and comprehensiveness of the detection results in the strategy combining QF-PCR and CNV-Seq, with cost being a secondary consideration. Even though Strategy 2 and 3 reduce costs, they inevitably miss some abnormalities or fail to detect maternal cell contamination. We believe that they are not worth recommending.

This study has several limitations. The inclusion of a small number of cases in the study may result in data bias. Additionally, we categorized variants of uncertain significance in CNVs as chromosomal abnormalities without considering the results of parental chromosome verification, leading to an overall higher detection rate of chromosomal abnormalities.

## 5 Conclusion

Our research revealed that the detection rate of chromosomal abnormalities in products of conception is as high as 67.20%, with chromosomal abnormalities being the primary cause of spontaneous abortion. The most common abnormality is aneuploidy. QF-PCR is effective in detecting maternal cell contamination, common aneuploidies, and triploidy, but it is not effective in detecting mosaicism. QF-PCR serves as a valuable complement to CNV-Seq. products of conception from women with advanced maternal age, a history of spontaneous abortion, early abortion, or natural conception are more likely to have chromosomal abnormalities. The use of QF-PCR on all samples to exclude maternal cell contamination before conducting CNV-Seq is the optimal detection strategy.

## Data Availability

The datasets presented in this article are not readily available because of ethical and privacy restrictions. Requests to access the datasets should be directed to the corresponding authors.
